# Blood Plasma Self-Separation Technologies during the Self-Driven Flow in Microfluidic Platforms

**DOI:** 10.3390/bioengineering8070094

**Published:** 2021-07-03

**Authors:** Yudong Wang, Bharath Babu Nunna, Niladri Talukder, Ernst Emmanuel Etienne, Eon Soo Lee

**Affiliations:** 1 Advanced Energy Systems and Microdevices Laboratory, Department of Mechanical and Industrial Engineering, New Jersey Institute of Technology, Newark, NJ 07102, USA; yw35@njit.edu (Y.W.); bn63@njit.edu (B.B.N.); nt22@njit.edu (N.T.); eee9@njit.edu (E.E.E.); 2 Division of Engineering in Medicine, Department of Medicine, Brigham and Women’s Hospital, Harvard Medical School, Harvard University, Cambridge, MA 02139, USA

**Keywords:** passive self-separation, microfluidics, microfiltration, sedimentation, Dean vortex, hydrophilicity

## Abstract

Blood plasma is the most commonly used biofluid in disease diagnostic and biomedical analysis due to it contains various biomarkers. The majority of the blood plasma separation is still handled with centrifugation, which is off-chip and time-consuming. Therefore, in the Lab-on-a-chip (LOC) field, an effective microfluidic blood plasma separation platform attracts researchers’ attention globally. Blood plasma self-separation technologies are usually divided into two categories: active self-separation and passive self-separation. Passive self-separation technologies, in contrast with active self-separation, only rely on microchannel geometry, microfluidic phenomena and hydrodynamic forces. Passive self-separation devices are driven by the capillary flow, which is generated due to the characteristics of the surface of the channel and its interaction with the fluid. Comparing to the active plasma separation techniques, passive plasma separation methods are more considered in the microfluidic platform, owing to their ease of fabrication, portable, user-friendly features. We propose an extensive review of mechanisms of passive self-separation technologies and enumerate some experimental details and devices to exploit these effects. The performances, limitations and challenges of these technologies and devices are also compared and discussed.

## 1. Introduction

**Figure 1:**
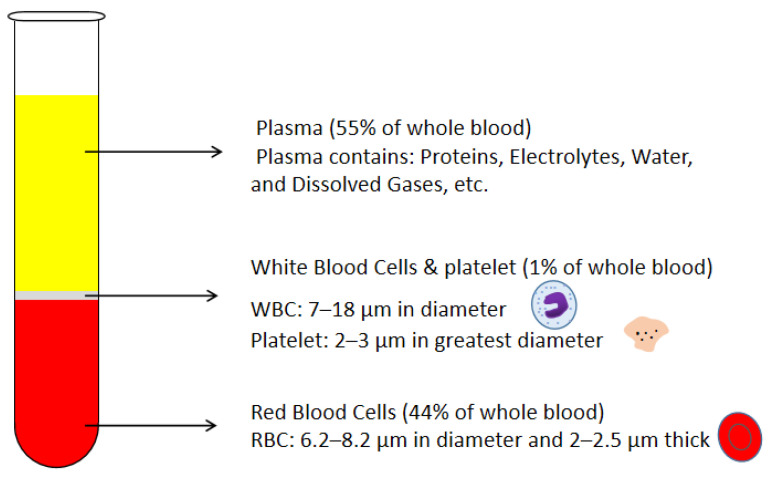
Schematic drawing of blood components and corresponding volumetric percentages in the whole blood.

**Figure 2:**
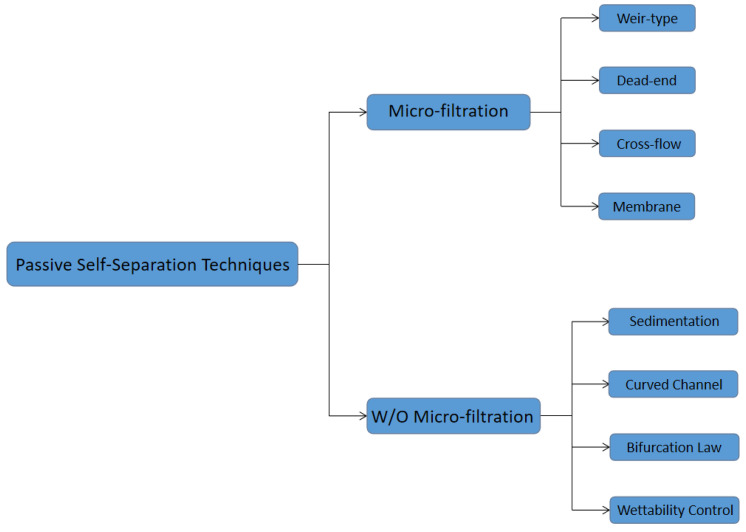
Schematic representation of the passive self-separation techniques discussed in this review paper.

Many recent advancements are developed in the Lab-on-a-chip field. However, the promotion of point-of-care (POC) technology is still an issue. POC devices take advantage of LOC technology and have a more comfortable, cheap, lab-free and convenient diagnosis process instead of the traditional laboratory process [1,2,3,4,5,6,7]. In the development of LOC technology, sample preparation is always an obstacle, especially in microfluidic platforms [8,9,10,11,12,13,14,15,16,17]. Blood is the most commonly used biofluid sample in LOC devices. There are many commercial applications that existed; lateral flow immunoassay applications; sandwich assays and competitive assays, vertical flow immunoassay application; antibody conjugation and screening, and paper microfluidic implementation; glucose detection, 3D devices for glucose detection and environmental and food safety tests [18,19,20,21,22,23]. Human whole blood comprises all blood components: red blood cells (RBCs), white blood cells (WBCs), platelet and plasma. Healthy human blood is around 55% plasma and 45% RBCs [8]. The sizes of each blood components are shown in Figure 1 [24,25,26]. When the blood sample is the analyte of LOC devices, which component of blood is desired should be considered. In many LOC applications, plasma is the desired analyte that contains the target biomarkers to be detected. For example, in ovarian cancer detection, CA-125 and HE-4 antigens are usually suspended in the plasma and are used as biomarkers in ovarian cancer diagnostics [27,28,29,30,31,32,33,34,35,36]. Furthermore, the viscosity of the blood sample affects the design of the microfluidic devices. The viscosity of blood varied with the patients’ sex, health conditions and diet. The viscosity of the whole blood mainly depends on the hematocrit. The higher the hematocrit of the whole blood, the more viscous the whole blood is. Temperature is another factor that affects the viscosity of the blood. There should be a temperature difference between the blood in the microchannel and blood in vivo. Additionally, the viscosity of the whole blood affects the flow rate of blood samples in the microfluidic channel. Therefore, the viscosity variation of different patients should also be considered in the microfluidic device design. The separation of plasma is the first step in most biomedical analysis assays. The standard separation process is centrifugation, as it is time-consuming and off-chip. It is not an ideal technique for microfluidic LOC devices [37]. Therefore, an effective and capillary-scale volume delivery system is expected to separate blood plasma and be integrated into the microfluidic sensing platform. Various separation technologies such as size-based particle sorting [38,39], active self-separation with external devices were concluded and discussed in many papers [40,41,42]. This paper is mainly focusing on the passive blood-plasma self-separation during capillary-driven flow in a microfluidic platform, Figure 2 shows the passive self-separation techniques discussed in this paper. In contrast with active self-separation techniques, passive blood-plasma self-separation does not need external forces or functionalities but is only driven by capillary flow enabled by a microchannel with hydrophilic walls [43,44,45,46,47,48,49,50]. Even though much research is carried out on developing passive blood-plasma self-separation systems in recent years, capillary-scale plasma preparation is still the bottleneck to the LOC technology. 

Various challenges are considered when developing a passive blood-plasma self-separation system. First of all, volume is limited in LOC devices. To ensure the comfort of patients, 1–2 μL of blood is the suggested volume of a finger prick [51]. With this small-scale blood sample, microchannel size is also limited. Due to this limitation, many hydrodynamic methods cannot have full effects on the plasma separation, such as the Dean vortex effect is limited by the low flow rate and low Reynolds number of the blood flow. A short separation time is also expected in the blood delivery systems. Usually, plasma is ideally separated and collected from whole blood in few minutes in the POC applications. With the long-time separation process, the whole blood has an increased risk of coagulation [52]. Clogging is another challenge, especially in the microfluidic channel with micro-filtration [53]. With continuous blood flow passing through the micro-filtration, blood cells accumulate in the filters and gradually block the micro-structures and stop the self-separation process. The throughput and purity of separated plasma are also included in the consideration [54]. Lastly, the integration of the blood delivery system and the sensing platform is a challenging problem. Numerous studies have been performed on sample preparation and integration and have been discussed for many years [55,56]. The integration process itself has its own difficulties, such as leakage problems, the complexity of disparate materials, etc. 

The fabrication processes of the microfluidic devices are mostly included in nanofabrication and have been mentioned by many papers. However, to have a capillary-driven flow in the microchannels, surface treatments are needed when the channel materials are naturally hydrophobic. There are many methods to change the hydrophilicity of the material surface. 1. Oxygen plasma treatment. The reactive species generated by oxygen plasma attacks the siloxane backbone of PDMS to form oxygen-rich SiOx silica-like layer and Si–OH surface structures [3]. 2. Nano particle deposition. There are many ways to deposit nano particles onto the material surface: Deposition by Electro-spinning, Spontaneous Growth on Surfaces, Deposition by Spray Coating, etc. For example, this paper mentioned that Lee et al. sprayed a layer-by-layer (LbL) nano-assembly layer on the top surface [57]. 3. Chemical etching. Immersion into the particular chemical can form a nanostructured layer on the material surface, changing the surface’s wettability [58].

Many reviews have focused on the micro-scale particle separation and overview of blood plasma separation [59,60,61,62,63]. This article focuses mainly on the passive blood plasma separation during the capillary-driven flow in the microfluidic platforms. This paper intends to critically review the recently developed advancements of the blood plasma self-separation mechanisms and applications in the self-driven flow. In the next sections, the mechanisms of passive self-separation with/without micro-structures will be categorized and discussed. Followed by a review of the currently available applications of passive blood-plasma self-separation technologies will be investigated. In this section, a comparison analysis is made to provide a better view of recent advances in the passive blood plasma self-separation technology in the LOC field. Subsequently, we will conclude and provide possible future directions for passive blood plasma self-separation in microfluidic applications.

## 2. Passive Self-Separation with Filtration Using Micro-Structures

### 2.1. Basic Mechanisms

**Figure 3:**
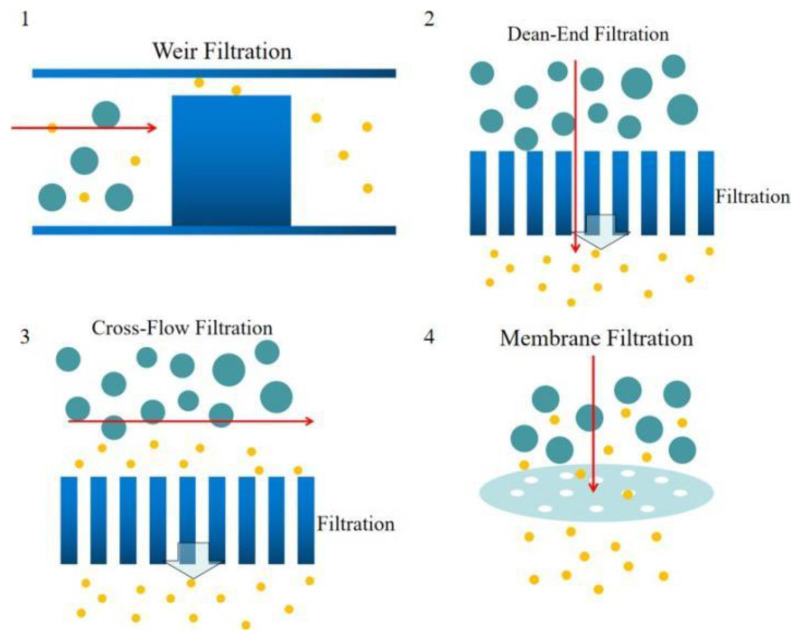
Schematics of the microfiltration types, (**1**) weir filtration (side view), (**2**) dead-end filtration (top view), (**3**) cross- flow filtration (top view), (**4**) membrane filtration (side view).

The blood plasma microfiltrations are usually categorized into four design types:1. Weir-type filtration 2. Dead-end pillar filtration 3. Cross-flow filtration, and 4. Membrane filtration [64]. A weir filter consists of obstacles that can obstruct the blood cells, and blood plasma can pass through the narrow slot on the top of the barrier [65,66]. The dead-end pillar microfiltrations involve a row of pillar structures with a critical spacing dimension to block the blood cells in the blood flow direction and extract blood plasma [67]. However, the clogging issues should always be considered in this type of microfiltration. In contrast with dead-end filtration, in cross-flow pillar microfiltration, the pillars are located perpendicular to the main blood flow, and the trapped blood cells will be flushed out of the filters by the main blood flow and avoid most clogging problems [68]. In the membrane microfiltrations, pores are located on a planar layer substrate. Feed blood flow is introduced into one side of the membrane, and the blood plasma will be extracted out to the other side of the membrane [69,70,71]. In the membrane filtration technique, pore sizes are more flexible but increase the complexity in the fabrication process. Figure 3 shows the schematic diagrams of four types of microfiltrations. 

### 2.2. Applications

#### 2.2.1. Weir-Type Filtration

**Figure 4:**
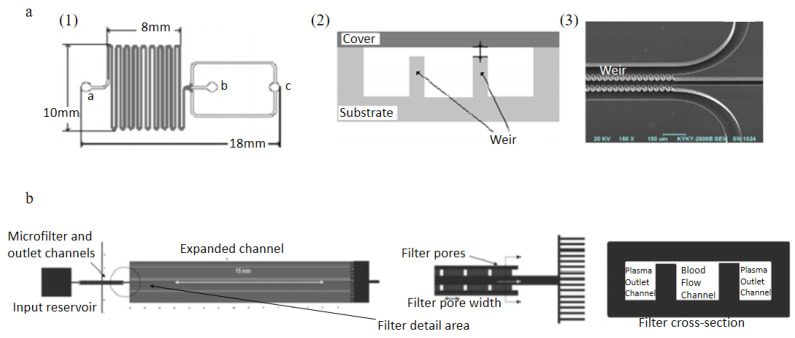
Applications of weir-type filtration, (**a**): schematic view of the silicon substrate, “a” is the inlet, “b” is the outlet for WBCs, “c” is the outlet for RBCs (1); cross-section of the weir-type barrier (2); SEM micrograph of the channel close to the outlet (3); (**b**) microfiltration design of Crowley et al. [72,73]. (**a**) reprinted from Sensors and Actuators B: Chemical, Volume 130, Issue 1, Xing Chen, Da Fu Cui, Chang Chun Liu, Hui Li, Microfluidic chip for blood cell separation and collection based on crossflow filtration, 216–221, Copyright (2007), with permission from Elsevier. (**b**) republished with permission of The Royal Society of Chemistry, from Lab on a Chip, Isolation of plasma from whole blood using planar microfilters for lab-on-a-chip applications, Timothy A. Crowley and Vincent Pizziconi, 5, 2005; permission conveyed through Copyright Clearance Center, Inc.

Chen et al. [72]. Designed cross- flow filtration microfluidic chips involved a PDMS- glass compounded cover and a silicon substrate, shown in Figure 4a. This device consisted of one inlet for introducing blood sample, and two outlets for collecting WBCs and RBCs, weir-type and pillar-type filtration barriers were designed in the microchannels, weir-type filtration is shown in Figure 4a and pillar-type filtration is shown in Figure 4a. The diluted blood samples were introduced into the device inlet in the experiments, and a variety of channel lengths were used as the separation length. As a result, 82.3% RBCs and 8% WBCs could be removed from the blood sample by the pillar-type filtration chip with 160 mm separation length, while 91.2% RBCs and 27.4% WBCs by weir-type filtration chip. A weir-based micro-filter using capillary action was designed by Crowley et al. [73]. In this device, human whole blood was introduced into a main blood flow channel connected with two lateral channels used for extracting plasma. Between main channel and lateral channels, weir-type barriers were evenly distributed to prevent blood cells flow into the lateral channels, shown in Figure 4b. Recently, multiple combinations of channel shapes and weir filtration structures were investigated to improve the separation yield and the purity of the extracted plasma [74,75].

#### 2.2.2. Dead-End Filtration

**Figure 5:**
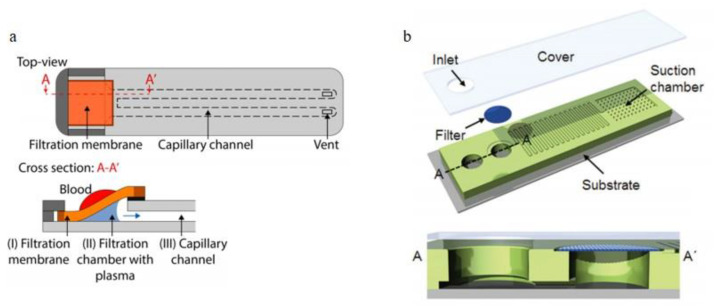
Applications of the dead-end filtration, (**a**) top-view and cross-section of Hauser et al.’s design, (I) a porous filtration membrane, (II) a filtration chamber and (III) the capillary channel; (**b**) microfiltration device integrated into a vertical-up flow channel, a membrane filter was positioned on the top of the vertical-up flow channel for filtration [76,77]. (**a**) reprinted with permission from Analytical Chemistry, High-Yield Passive Plasma Filtration from Human Finger Prick Blood, Janosch Hauser, Gabriel Lenk, Jonas Hansson, Olof Beck, Göran Stemme, and Niclas Roxhed, 2018, 90, 22, 13393–13399. Copyright (2018) American Chemical Society. Figure 5b republished with permission of the Royal Society of Chemistry, from Lab on a Chip, Hemolysis-free blood plasma separation, Jun Ho Son, Sang Hun Lee, Soongweon Hong, Seung-min Park, Joseph Lee, Andrea M. Dickey and Luke P. Lee, 14, 2287, 2014; permission conveyed through Copyright Clearance Center, Inc.

Dead-end filtrations are usually used to process small amounts of blood samples to avoid clogging issues. Hauser et al. [76]. developed a dead-end membrane microfiltration device contains: a porous filtration membrane for blood plasma separation, a capillary microchannel for extracting plasma and a filtration chamber connecting membrane and capillary channel, details are shown in Figure 5a. 13–21 μL of plasma was extracted from 50 μL of blood sample within the hematocrit range of 35–55%, and a high extraction yield of 65% was achieved within less than 10min. Son et al. [77]. Reported a microfluidic blood plasma separation device with commercially used track-etched polycarbonate membrane filters with 4 μm pores for plasma separation, the schematic illustration of the device is shown in Figure 5b. The membrane filtration was placed on the top of the vertical up-flow channel, and gravity prevented the clogging of the filtration. By using this device, up to 4 μL of separated blood was extracted from the 30% hematocrit feeding blood. Both these two devices had limitations in separating plasma from high hematocrit blood samples effectively.

#### 2.2.3. Cross-Flow Filtration

**Figure 6:**
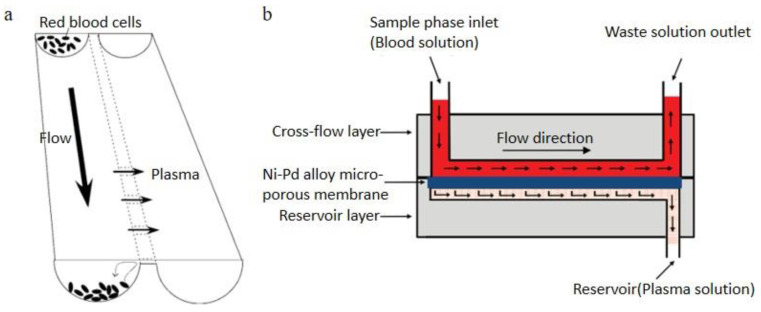
Cross-flow filtration, (**a**) principle of plasma separation from whole blood in the microchannel; (**b**) schematic of Yeh et al.’s cross-flow filtration method [78,79]. (**a**) reprinted with permission from Analytical Chemistry, Simultaneous Separation, Metering, and Dilution of Plasma from Human Whole Blood in a Microfluidic System, Tomoya Tachi, Noritada Kaji, Manabu Tokeshi, and Yoshinobu Baba, 2009, 81, 3194–3198. Copyright (2009) American Chemical Society. (**b**) republished with permission of IOP Publishing, Ltd, from Journal of Micromechanics and Microengineering, Using the developed cross-flow filtration chip for collecting blood plasma under high flow rate condition and applying the immunoglobulin E detection, Chia-Hsien Yeh, Chia-Wei Hung, Chun-Han Wu and Yu-Cheng Lin, 24, 095013, 2014; permission conveyed through Copyright Clearance Center, Inc.

Cross-flow filtration technology can effectively avoid clogging issues and be widely studied in the POC field. Tachi et al. [78]. Demonstrated a cross-flow filtration micro-device consisted of two main parallel microchannels connected by multiple shallow channels. Shallow channels were 12 μm wide and 1 μm deep. A schematic diagram is shown in Figure 6a. Using this device, plasma could be extracted from whole blood then be metered and diluted simultaneously without hemolysis. Yeh et al. [79]. Presented a cross- flow filtration chip for extracting plasma from whole blood, which includes a cross- flow layer, a Ni-Pd alloy micro-porous membrane and a reservoir layer, shown in Figure 6b. The comparative experiments were completed using various diluted blood samples, membranes with different pore sizes and different flow rates. 96.2% separation efficiency was finally achieved as the best result from the 10× diluted blood sample with 2 μm pore size Ni-Pd alloy micro-porous membrane. Some researchers utilize the deformability of the RBCs and separate RBCs in the cross-flow filtration [80]. To additionally avoid rapid irreversible clogging, a reversal flow was applied in the crossflow filtration [81]. 

#### 2.2.4. Membrane Filtration

**Figure 7:**
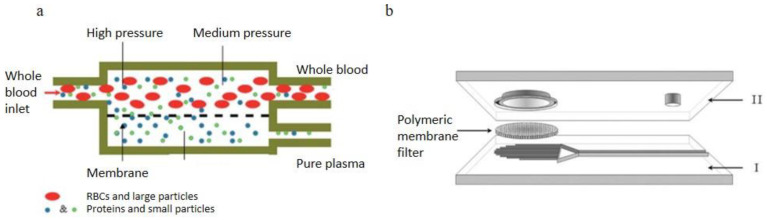
Membrane filtration, (**a**) Schematic of the two-compartment microfiltration device, the top compartment is the main flow channel consists of all blood components and the plasma flow across the permeable membrane into the bottom compartment. (**b**) diagram of the hybrid filtration device consists of a lid, a 13 mm diameter polymeric membrane filter and a bottom substrate with 40 parallel microchannels that collect filtrate and lead it into the reservoir [82,83]. (**a**) republished with permission of the Royal Society of Chemistry, from Lab on a Chip, Microfiltration platform for continuous blood plasma protein extraction from whole blood during cardiac surgery, Kiana Aran, Alex Fok, Lawrence A. Sasso, Neal Kamdar, Yulong Guan, Qi Sun, Akif Ündar and Jeffrey D. Zahn, 11, 2858–2868, 2011; permission conveyed through Copyright Clearance Center, Inc. (**b**) Reprinted by permission from Springer Nature Customer Service Centre GmbH: Springer, Biomedical Microdevices, A hybrid poly(dimethylsiloxane) microsystem for on-chip whole blood filtration optimized for steroid screening, Sara Thorslund, Oliver Klett, Fredrik Nikolajeff, Karin Markides, and Jonas Bergquist, 8, 73–79, Springer-Verlag Berlin Heidelberg 2006 (2006).

Aran et al. [82]. reported a membrane micro-filtration device using cross-feeding blood flow, the membrane filtration was sandwiched between two PDMS microchannels as Figure 7a. Two PDMS layers were adhered by surface modification of the polymer membrane via 3-aminopropyltriethoxysilane (APTES). They experimented with the sheep blood with various HCT levels (42%, 34%, 30% and 20%), the results showed that the separation rate decreased with experiment time when using the 42% HCT sheep blood. However, over the duration of the experiments using 20% to 34% HCT blood samples, the blood plasma separation was effective. Thorslund et al. [83]. reported a device with hydrophilic polypropylene (PP) membrane filter integrated between two PDMS slabs, shown in Figure 7b. The membrane filter had 0.4–0.45 μm pores. As addressed by Thorslund, the limitation of this device is the RBCs leakage caused by too high lid structure, and the leakage issue could not be significantly improved by sealing. Therefore, the best solution to improve this microsystem is to handle as large blood volumes as possible. Devices with the high lid design would face the same issues. Only diluted blood under 20% hematocrit (Hct) could be used to avoid this problem. This device ran on a PP device to prevent hemolysis and blood cell leakage in the separation process.

## 3. Passive Self-Separation without Filtration

### 3.1. Mechanisms

#### 3.1.1. Dean Flow Fractionation

Due to the complicated balance of hydrodynamic forces, particles suspended in the microfluidic flow could be sorted by their sizes and densities. In a spiral microchannel, the flow experiences a centrifugal force when passing through the curved channel. The fluid on the outer side of the channel has a relatively higher pressure and forms a pressure gradient toward the center of the curvature of the curved channel. In a viscous flow, the velocity profile is not uniform. The velocity near the channel wall is lower than in the center of the channel and causes a lower centrifugal force on the outer channel wall. This will cause a secondary flow from the channel center to the outer channel wall. Meanwhile, due to the pressure gradient from the outer side to the curvature center, a flow will flow along the channel wall from the convex wall to the concave wall. These secondary flows formed a pair of the symmetric vortex is called Dean vortices [84,85].

**Figure 8:**
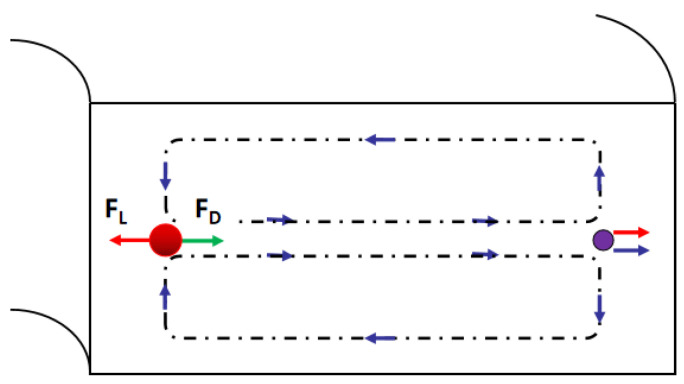
Schematic illustrating the effect of the curvature, the larger particles focus on the position closer to the inner channel wall due to the dean vortex.

Particles suspended in the spiral microchannel experience the inertial lift force caused by the microchannel cross-section and the dean drag force due to the Dean vortices, shown in Figure 8. Inertial lift force:

*F_L_* = *ρG*^2^*C_L_a*^4^*_p_* (1)

where *ρ* is the fluid density, *G* is the shear rate, *C_L_* is the lift coefficient, *a_p_* is the diameter of the particle. The Dean drag force:

*F_D_* = *3πµU_D_ a_p_* (2)

where *μ* is the viscosity of the fluid, U_D_ is the average Dean velocity *U_D_* = 1.8 × 10^4^ De^1.63^ (De is Dean number De = Re(D_h_/2R)^0.5^, Re is Reynolds number, D_h_ is hydraulic diameter, R is the radius of the curvature of the convex channel wall). The particles or cells will migrate to a focus position when the dean drag force and the inertial lift force are balanced [86].

#### 3.1.2. Mechanisms and Limitations of Sedimentation Technology

**Figure 9:**
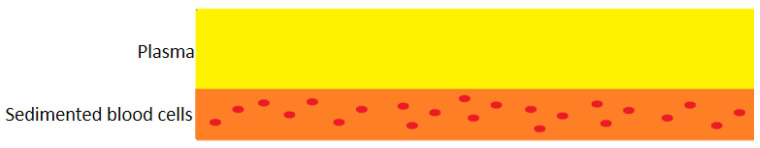
Schematic of sedimentation technique.

Sedimentation is one of the oldest separation approaches based on the gravity and the density differences between plasma and blood cells (*ρ*_RBCs_=1100 kg/m^3^, *ρ*_WBCS_= 1050–1090 kg/m^3^ and *ρ*_plasma_=1030 kg/m^3^), the principle is shown in the Figure 9. Sedimentation velocity of blood cells in the whole blood is varied with patients’ sex and health conditions [87]. The biggest limitation of sedimentation technology is the low separation velocity, and it is out of consideration when facing a large volume of the blood sample. However, with a small amount of blood, such as finger prick, it is still a popular technology. Hybridizing the sedimentation technique with filtration advantageously minimizes clogging issues inherent to microfiltration [88,89]. Most blood cells sediment before reaching the filtration region, the plasma could be continuously extracted and a few suspension blood cells are separated away by the filter.

#### 3.1.3. Bifurcation Law (Zweifach-Fung Effect)

**Figure 10:**
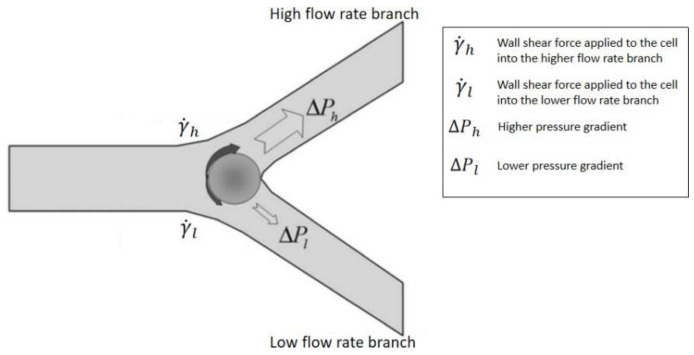
Zweifach-Fung effect schematic diagram, when the flow rate ratio is more than 2.5, and the cell-to-vessel diameter ratio is of the order of 1. Red blood cells tend to flow into the higher flow rate daughter vessel instead of, the lower flow rate daughter vessel due to the pressure difference and shear forces acting on the cell [91]. Republished with permission of the Royal Society of Chemistry, from Lab on a Chip, A microfluidic device for continuous, real time blood plasma separation, Sung Yang, Akif Ündar and Jeffrey D. Zahn, 6, 871-880, 2006; permission conveyed through Copyright Clearance Center, Inc.

Bifurcation law also called the Zweifach-Fung effect, describes when the blood flowing in the capillary and passing through a bifurcating region, the RBCs in the blood flow into the higher flow rate daughter vessel. In contrast, only a few RBCs flow into the lower flow rate daughter vessel [90]. This effect will occur when the flow rate ratio between two daughter channels is above the critical ratio: 2.5:1. The diameter of the channel wall should not be too much larger than the diameter of cells [91]. As Figure 10 shows, at the bifurcation region, the asymmetric shear forces are applied on the cell’s surface due to the flow rate difference and produce a torque to pull it into the high flow rate channel [92].

#### 3.1.4. Microchannel Surface Control of Wettability

**Figure 11:**
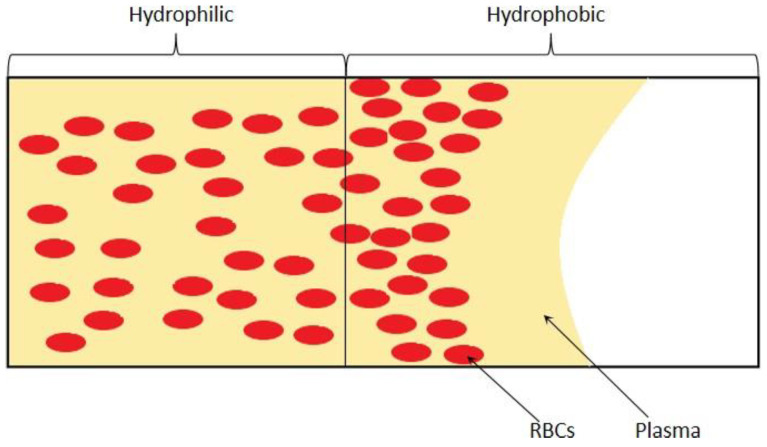
Schematic of surface control of wettability.

The passive self-separation microfluidic channel’s blood flow is driven by capillary force induced by hydrophilic channel walls [93]. Researchers designed a microchannel with different hydrophilicity patterns to separate plasma based on this principle, as can be appreciated in the Figure 11 that shows. Asymmetric channel with 3 sides hydrophilic in the front part of the channel and a hydrophobic patch with all walls hydrophobic is applied followed with the hydrophilic part. When the blood sample is introduced into the channel inlet, with the hydrophilic channel walls and Young-Laplace pressure, the blood flow into the microchannel as a capillary-driven flow. With the large contact angle and the inverse direction Young-Laplace pressure, the blood flow is impeded at the hydrophobic region. Since the viscosities of blood plasma and blood cells are different, the plasma has a higher velocity in the hydrophobic patch than the blood cells and passes through the hydrophobic region. Meanwhile, the blood cells stop and accumulated. In this technique, the asymmetric hydrophilic channel walls provide a gentler velocity decrease when the whole blood flow downstream [94], and the velocity could be controlled by the contact angle of the channel walls [95].

### 3.2. Self-Separation of Blood Plasma during the Self-Driven Flow in Micro-Devices

#### 3.2.1. Sedimentation Applications

**Figure 12:**
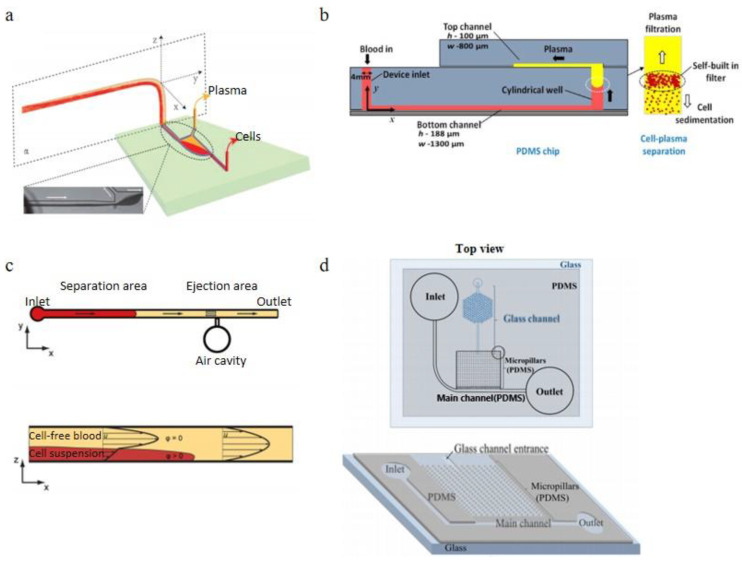
Sedimentation Applications, (**a**) schematic for the principle of the plasmapheresis device; (**b**) schematic of the device structure, plasma extraction due to the sedimentation and self-build-in filter; (**c**) two device structures: separation and ejection, and the illustration of the separation principle; (**d**) top view (top) and 3D schematics (bottom) of Park et al.’s blood separator [96,97,98,99]. (**a**) reprinted with permission from Analytical Chemistry, Gravitational Sedimentation Induced Blood Delamination for Continuous Plasma Separation on a Microfluidics Chip, Xian-Bo Zhang, Zeng-Qiang Wu, Kang Wang, Jie Zhu, Jing-Juan Xu, Xing-Hua Xia, and Hong-Yuan Chen, 2012, 84, 8, 3780–3786. Copyright (2012) American Chemical Society. (**b**,**c**) reprinted from [96,97] under the terms of the Creative Commons CC BY license. (**d**) reprinted by permission from Springer Nature Customer Service Centre GmbH: Springer, Microsystem Technologies, 22, 2077–2085 (2016), On-chip whole blood plasma separator based on microfiltration, sedimentation and wetting contrast, Sanghoon Park, Roxana Shabani, Mark Schumacher, Yoon-Seoung Kim, Young Min Bae, Kyeong-Hee Lee, Hyoung Jin Cho, Springer-Verlag Berlin Heidelberg 2015, (2015).

Zhang et al. [96]. reported a continuous plasma extraction microfluidic device, their method is to keep the sedimentation of erythrocytes unperturbed in the glass capillary and microchannel, shown in Figure 12a. The innovative part of this design is the orientation changing of the connector, which enhanced the separation efficiency. The purity of this design could achieve 99% with 1:5 diluted blood (8% Hct) within the feed flow rate of 30 μL/min. As the feed flow rate increases, more sample consumption is caused by the shorter sedimentation time and more turbulence at the connector and decreases the separation efficiency. There is no clogging observed in 4 hours separation time. A double layer PDMS microchannel blood-plasma separator with wettability gradient and sedimentation effect was demonstrated by Maria et al. [97]. shown in Figure 12b. A vertical cylindrical well connected the top microchannel and the bottom microchannel with a wettability gradient, the center portion of the well is more hydrophobic, and the two sides of the well are more hydrophilic, which can enhance the plasma separation by the velocity difference between blood cells and plasma when the blood sample passing through the well. By this combined gravity and capillarity, the plasma was separated from the whole blood. In this experiment, 2.0 μL of plasma was separated from less than 10 μL whole blood in 15 min with 99.9% purification efficiency. Forchelet et al. [98]. have recently developed a micro-device to separate plasma from whole blood using capillary flow and sedimentation effect shown in Figure 12c. The blood cells sediment toward the bottom of the microchannel due to the gravity, and the blood delamination occurred based on the viscosity differences between blood cells and plasma. In this design, there was an ejection area following the separation portion of the channel. With this area, the volume of the cell-free blood sample can be measured. This device achieved a 99.987% purity in plasma separation. Sedimentation technology can advantageously avoid clogging issues when it is in combination with micro-filtration, Park et al. [99]. demonstrated a passive blood separator with a combined design strategy including micro-filtration, sedimentation and wettability gradient. This device was composited by an etched glass hydrophilic microchannel bottom layer and a hydrophobic natural PDMS top layer with a micropillar array, shown in Figure 12d. The separation efficiency was near 100%, and a small volume of blood (<15 μL) was required in this device.

#### 3.2.2. Curved Channel Applications

**Figure 13:**
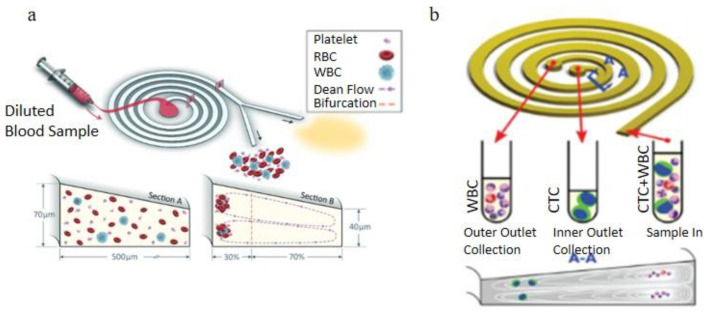
Spiral microchannel with trapezoidal cross-section, (**a**) schematic of the blood plasma separation using a spiral channel with a trapezoidal cross-section, blood cells concentrated near the inner channel wall around the vortex cores; (**b**) The operating principle of blood cells separation by a spiral channel with a trapezoid cross-section, CTCs focused near the inner channel wall and WBCs are trapped closer to the outer channel wall [101,102]. (**a**) republished with permission of the Royal Society of Chemistry, from Lab on a Chip, Multiplexing slanted spiral microchannels for ultra-fast blood plasma separation, Mehdi Rafeie, Jun Zhang, Mohsen Asadnia, Weihua Li and Majid Ebrahimi Warkiani, 16, 2791–2802, 2016; permission conveyed through Copyright Clearance Center, Inc. (**b**) reprinted from [101] under the terms of the Creative Commons CC BY license.

In the spiral microchannel, the cross-section of the channel is found to be another factor that could enhance the Dean vortex effect in the blood plasma separation process [100]. Some researchers advantageously used the trapezoidal cross-section of the spiral microchannel to make the cores of Dean vortices migrate to the longer side of the channel and trap larger particles in the center of the Dean vortices additionally sort out suspended particles by their sizes. Rafeie et al. [101]. demonstrated a spiral microchannel with the trapezoidal cross-section by which can enhance the cells focusing abilities in the microchannel. With low concentrated blood samples (0.5% and 1% Hct), they achieve the 100% purity of separated plasma under a 1.5 mL/min flow rate. However, with the input blood concentration increasing, the separation efficiency of this device significantly decreases. They additionally designed an innovative multiplexing spiral channels device for ultra-fast blood plasma separation, and each microchannel has the same design as the proposed one, shown in Figure 13a. This multiplexing spiral channel can process 1mL of whole blood within 1min and is expected as a continuous and high throughput blood plasma separator. Warkiani et al. [102]. demonstrated a spiral microchannel with trapezoidal cross-section consists of one inlet and two outlets to separate the WBCs and the circulating tumor cells (CTCs), shown in Figure 13b. Their works improved the sorting purity (100%) for CTCs from patients with advanced-stage metastatic breast and lung cancer in the rapid blood processing speed. The two devices above were using different flow directions. With the radius of the microchannel’s curvature increased, Dean’s number decreased exponentially, weakening the separation efficiency.

**Figure 14:**
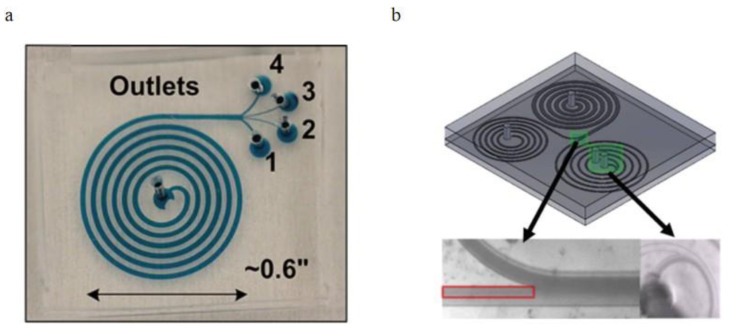
(**a**) Brightfield images of the microchannel, the flow boxed in red shows the cells passing the first bifurcation and to be filtered again in the second spiral channel, and the bottom right figure shows the second bifurcation and blood cells collected in the waste outlet; (**b**) schematic of the 3 outlets spiral channel, larger particles are collected in the inner outlet while most smaller particles flow into the middle outlet, and plasma are extracted into the outer outlet [86,103]. (**a**) reprinted from Biomicrofluidics, Continuous separation of blood cells in spiral microfluidic devices, Nivedita Nivedita and Ian Papautsky, 7, 5, 054101 (2013) with the permission of AIP Publishing. (**b**) reprinted from Biomicrofluidics, Rapid isolation of blood plasma using a cascaded inertial microfluidic device, M. Robinson, H. Marks, T. Hinsdale, K. Maitland, and G. Cotéwiththe, 11, 024109 (2017) with the permission of AIP Publishing.

Nivedita et al. [86]. developed a spiral microchannel with four outlets to sort the WBCs and the RBCs based on their sizes difference, shown in Figure 14a. Due to the larger size, the WBCs were closer to the inner side of the channel wall, and the RBCs were focusing near the outer wall when introducing the 500× diluted blood into the channel inlet. With the highly diluted blood, the separation process could avoid the strong cell to cell interaction and experience a larger effect of Dean vortices. Still, it was also a limitation when this device was applied to commercial use. They successfully received around 95 ± 2.2% of WBCs and 6 ± 2.4% of RBCs in the first outlet, and around 95% RBCs in the second and third outlets. The fourth outlet only had the plasma and the platelets. Further experiments were also accomplished using multiple inlet blood samples with various blood cells concentration. Instead of changing the cross-section of the microchannel, adding channel downstream length is also considered by researchers. Robinson et al. [103]. reported a micro device contains the main spiral microchannel followed by two secondary spiral microchannels. The function of the two secondary microchannels is to additionally filter out the blood cells with a similar flow velocity as the main microchannel, shown as Figure 14b. They finished the experiments with 2% Hct diluted blood and received 99% separation efficiency with the device with secondary spiral microchannels and 55% efficiency with the single main spiral microchannel. A recent beehive-like blood plasma separator with a separation unit contains many sub-spiral channels were designed to separate plasma based on the same principle [104].

#### 3.2.3. Applications of Bifurcation Law

**Figure 15:**
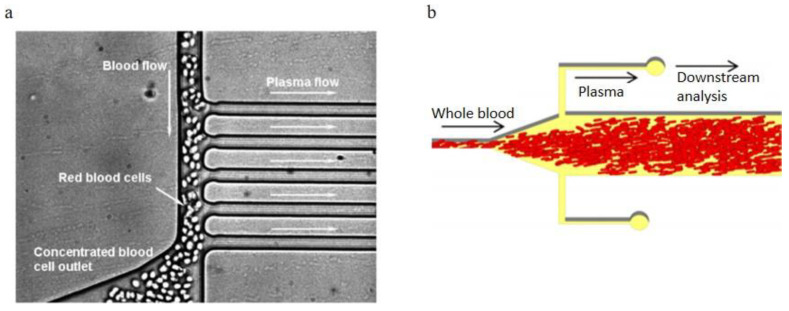
Bifurcation law applications, (**a**) A zoomed-in view of the blood plasma separation region, the main blood channel width is 15 mm, while all plasma skimming channels have widths of 9.6 mm; (**b**) Schematic of Shatova’s blood plasma separation device design [91,105]. (**a**) republished with permission of the Royal Society of Chemistry, from Lab on a Chip, A microfluidic device for continuous, real time blood plasma separation, Sung Yang, Akif Ündar and Jeffrey D. Zahn, 6, 871–880, 2006; permission conveyed through Copyright Clearance Center, Inc. (**b**) reprinted with permission from Analytical Chemistry, Portable, Constriction–Expansion Blood Plasma Separation and Polymerization-Based Malaria Detection, Tatyana A. Shatova, Shefali Lathwal, Marissa R. Engle, Hadley D. Sikes, and Klavs F. Jensen, 2016, 88, 15, 7627–7632. Copyright (2016) American Chemical Society.

Yang et al. [91]. took advantage of the Zweifach-fung effect to develop a microfluidic device consists of the main blood channel (width is 15 μm) and multiple plasma skimming channels (width is 9.6 μm), shown in Figure 15a. In their experiments, 10–35% Hct sheep blood samples were infused into the channel inlet, and 100% purity of extracted plasma was received with 4 μl/min extraction rate in the outlets. The volume of extracted plasma percent out of total plasma varied from 15% to 25% dependent on the hematocrit values of the inlet blood. Shatova et al. [105]. reported a constriction-expansion microfluidic blood plasma separator which can extract 100% pure plasma from whole blood at 9% yield. As shown in Figure 15b, blood flow first went through the constricted portion of the microchannel, then went downstream to the expansion area, which has two plasma skimming channels on both sides. Due to the Zweifach-fung effect, blood cells stayed in the main blood flow and plasma went into the two daughter microchannels at the bifurcation region. They also experimented with blood samples with various blood cells concentration in the microchannels with various expansion angles. Details were discussed in their article.

#### 3.2.4. Microchannel Wettability Control Separation Methodologies

**Figure 16:**
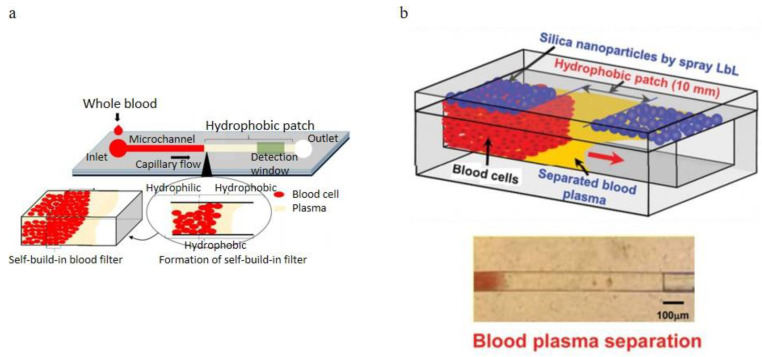
Wettability gradient effect applications, (**a**) Schematic of the capillary flow-driven blood plasma separation microchannel with a hydrophobic patch; (**b**) design of the microfluidic blood plasma separation channel with hydrophilicity gradient (top) and experimental magnified view of the separated plasma (bottom) [57,95]. (**a**) reprinted from Biomicrofluidics, Capillary flow of blood in a microchannel with differential wetting for blood plasma separation and on-chip glucose detection, M. Sneha Maria, P. E. Rakesh, T. S. Chandra, and A. K. Sen, 10, 054108 (2016) with the permission of AIP Publishing. (**b**) republished with permission of the Royal Society of Chemistry, from Lab on a Chip, A new on-chip whole blood/plasma separator driven by asymmetric capillary forces, Kang Kug Lee and Chong H. Ahn, 13, 3261–3267, 2013; permission conveyed through Copyright Clearance Center, Inc.

In self-driven flow microfluidic devices, modified channel surfaces could provide a velocity gradient of blood flow due to their viscosity difference, separating the blood cells and the plasma. Maria et al. [95]. designed a passive self-separation microfluidic channel with different hydrophilicity on its surface. As shown in Figure 16a, they added a hydrophobic patch after the hydrophilic surface-treated microchannel. Since the viscosities difference of plasma and the blood cells are significant, plasma had a higher flow velocity than the blood cells in the hydrophilic channel. Therefore the plasma could pass through the hydrophobic region meanwhile the blood cells stopped. Thus, plasma successfully separated from the whole blood. In their approach, 450 nL plasma could be extracted in 15 min and the purity of the plasma was comparable with that obtained using the centrifugation process. They also provided experimental data for the microchannels with various surface contact angles. They finished the comparison experiments on the all-wall hydrophilic and asymmetric (bottom is hydrophobic and other walls are hydrophilic) microchannel. Lee et al. [57]. used the microfluidic channel driven by asymmetric capillary flow. The microchannel was made of cyclic olefin copolymer (COC), which was naturally hydrophobic. To control the asymmetric capillary flow in the microchannel, a spray layer-by-layer (LbL) nano-assembly layer was sprayed on the top surface, which was supposed to be super hydrophilic but left a hydrophobic region. All other three walls (the bottom and two sides walls) were untreated and kept hydrophobic, as shown in Figure 16b. By comparing the microchannels with different hydrophobic patch lengths and channel widths, a maximum of 100 nL plasma was separated from the device with the 100 μm width and 10 mm hydrophobic patch.

From Table 1, sedimentation and bifurcation effect techniques could deal with undiluted whole blood samples and achieved good separation efficiencies, but the separated plasma amounts are limited [106,107,108,109,110,111,112,113,114]. Dean vortex effect methods could only process the diluted blood samples due to the limitation of viscosity and velocity of the fluid in the spiral microchannel, although they have good efficiencies and short separation times [115,116,117,118,119,120,121,122]. In wettability control technologies, pure plasma could be extracted from whole blood successfully, but separation time is comparably long, and surface modification is a complex process in microchannel fabrication [123,124,125,126,127].

## 4. Discussion and Future Direction

This review has primarily focused on the passive blood-plasma self-separation techniques, emphasized the basic mechanisms of passive separation methods without micro-filtration and introduced novel applications based on these theories. The passive blood-plasma self-separation technique is a possible candidate for LOC applications and overcomes many blood plasma separation challenges. For instance, the passive self-separation techniques can effectively avoid complex fabrications and easy to use in combination to overcome each other’s limitations. However, in the POCT applications, the exact sample provided by patients will restrict the separation methodologies. For example, the techniques based on the dean vortex are hard to satisfy the situation which required finger-prick amount of blood sample because the channel volumes are usually larger than finger-prick blood sample volumes. However, sedimentation technology with capillary flow actuation is fit for this application. To separate capillary volumes of blood, slow separation rates could be negligible compared to their performance. Additionally, the micro-filtration structures integrated into the sedimentation device could improve the clogging issue and increase the volumes of extracted plasma. 

Even though numerous novel devices have been developed based on various technologies, nearly none has been considered commercially. The LOC community has been searching for effective and efficient microfluidic plasma separators in the past years, especially which could process undiluted blood. Yet, they have not been very successful until now. The viability of commercialization of microfluidic separation devices is affected by many aspects, such as the material choice for the microfluidic platform, the difficulty of the large scale of fabrication with PDMS, the reliable surface treatment process, commercial packaging should also be considered. Another limitation of the POC device commercialization is the patient’s operation of the devices. As a non-professional, patients are hard to precisely acquire the required amount of blood from finger prick as instructed. In the at-home sampling process, anti-coagulation of blood should also be considered [128]. Non-medical trained individuals usually do not know the coagulation process of the extracted blood. The anticoagulants should be precoated in the blood collection devices depending on the purpose of the POC devices. Additionally, they often lack an understanding of quality control. The misoperation may lead to inaccurate results. The sample volumes of blood plasma separation are categorized depending on the different purposes. For the small amount of blood from finger-pricks, sedimentation with micro-filtration could be first considered, but hydrodynamic techniques may not have a good performance. In contrast, to process a large sample volume of hundreds of micro liters, hydrodynamic techniques could address multiple challenges of other methods. Furthermore, a non-diluted blood sample is desired in commercial use, especially in POCT applications. However, most current techniques use diluted blood samples and the separation efficiency increases with the blood dilution level. Therefore, to use undiluted blood in microfluidic devices, a better understanding of blood flow in the microchannel is needed. The designers can have a better overview and abilities to improve the current technologies and promote the commercial use of the passive plasma separation platform. The future of the field of blood plasma separation is in the trend of hybridization of technologies. By combining techniques and taking advantage of distinct effects, designers can overcome challenges based on their design purposes.

## Figures and Tables

**Table 1:** Provides the features of various passive self-separation devices developed by different researchers.

Research Group	Design Principle	Blood Sample	Separation Efficiency %
Zhang et al. [96]	Sedimentation	8% Hct blood	99
Maria et al. [97]	Sedimentation	whole blood	99
Forchelet et al. [98]	Sedimentation	whole blood	99
Park et al. [99]	Sedimentation	whole blood	100
Rafeie et al. [101]	Dean vortex effect	0.5 and 1% Hct blood	100
Robinson et al. [102]	Dean vortex effect	2% Hct blood	99
N. Nivedita et al. [86]	Dean vortex effect	Diluted blood	95
Warkiani et al. [103]	Dean vortex effect	20–25% Hct blood	85
Yang et al. [91]	Bifurcation effect	Sheep whole blood	100
Shatova et al. [105]	Bifurcation effect	whole blood	100
Maria et al. [95]	Wettability control	whole blood	N/A
Lee et al. [57]	Wettability control	whole blood	N/A

## Data Availability

Not Applicable.
